# Endoplasmic Reticulum Stress-Mediated Cell Death in Renal Fibrosis

**DOI:** 10.3390/biom14080919

**Published:** 2024-07-28

**Authors:** Shangze Guo, Yinghao Tong, Ting Li, Kexin Yang, Wei Gao, Fujun Peng, Xiangyu Zou

**Affiliations:** School of Basic Medical Sciences, Shandong Second Medical University, Weifang 261053, China; 20230112@stu.wfmc.edu.cn (S.G.); 20230109@stu.wfmc.edu.cn (Y.T.); 20220115@stu.wfmc.edu.cn (T.L.); 20220118@wfmc-edu.cn (K.Y.); gaowei@wfmc.edu.cn (W.G.); pengfujun@wfmc.edu.cn (F.P.)

**Keywords:** endoplasmic reticulum stress, renal fibrosis, cell death

## Abstract

The endoplasmic reticulum (ER) is indispensable for maintaining normal life activities. Dysregulation of the ER function results in the accumulation of harmful proteins and lipids and the disruption of intracellular signaling pathways, leading to cellular dysfunction and eventual death. Protein misfolding within the ER disrupts its delicate balance, resulting in the accumulation of misfolded or unfolded proteins, a condition known as endoplasmic reticulum stress (ERS). Renal fibrosis, characterized by the aberrant proliferation of fibrotic tissue in the renal interstitium, stands as a grave consequence of numerous kidney disorders, precipitating a gradual decline in renal function. Renal fibrosis is a serious complication of many kidney conditions and is characterized by the overgrowth of fibrotic tissue in the glomerular and tubular interstitium, leading to the progressive failure of renal function. Studies have shown that, during the onset and progression of kidney disease, ERS causes various problems in the kidneys, a process that can lead to kidney fibrosis. This article elucidates the underlying intracellular signaling pathways modulated by ERS, delineating its role in triggering diverse forms of cell death. Additionally, it comprehensively explores a spectrum of potential pharmacological agents and molecular interventions aimed at mitigating ERS, thereby charting novel research avenues and therapeutic advancements in the management of renal fibrosis.

## 1. Introduction

The endoplasmic reticulum (ER) is an organelle in eukaryotic cells consisting of an intricate set of interconnected membranes and channels that form a complex network of tubes separated from the cytoplasmic matrix [1]. When disease occurs, the intracellular protein homeostasis is disrupted and many misfolded proteins accumulate in the ER, triggering endoplasmic reticulum stress (ERS) [2,3]. There are three main ERS sensors, namely inositol-requiring enzyme 1 (IRE1), protein kinase R-like endoplasmic reticulum kinase (PERK), and activating transcription factor 6 (ATF6). Under normal conditions, these sensors—along with immunoglobulin heavy-chain binding protein (BiP)/glucose-regulated protein 78 kDa (GRP78), an ER molecular chaperone—remain inactive [4,5]. Under ERS conditions, cells activate a complex network of mechanisms to determine their survival [6]. Renal fibrosis is a pathological complication of chronic kidney disease (CKD) and a typical feature of end-stage disease. Its morphology is characterized by glomerulosclerosis and vascular stenosis [7,8]. During kidney injury, local fibroblasts and peripheral cells are activated, contractility is increased, inflammation-associated chemicals are released, and extracellular matrix (ECM) components that promote wound repair are produced. However, if the injury is repeated or worsened, ECM proteins continue to accumulate in the kidney, which can lead to tissue destruction, kidney dysfunction, and, ultimately, organ failure [9]. The activation of ERS is closely associated with kidney disease. Studies have shown that ERS activates renal interstitial cells and promotes renal fibrosis [10].

## 2. Endoplasmic Reticulum and Endoplasmic Reticulum Stress

The ER is the largest organelle in the cell and is responsible for regulating many key physiological functions, including overseeing intracellular protein synthesis, maintaining calcium ion homeostasis, and producing steroids and other lipids [11]. The ER relies on a variety of regulatory molecules, such as molecular chaperones, glycosylation enzymes, sulfotransferases, transporters, and calcium ion channels [12]. During ERS, the unfolded protein response (UPR) is activated. The UPR reduces protein production, removes misfolded or unfolded proteins, and enhances the capability for protein folding within the ER. This response is mediated by three primary sensors, namely ATF6, PERK, and IRE1 [13,14]. Cytosolic ATF6, X-box binding protein 1 (XBP1), and activating transcription factor 4 (ATF4) work together to initiate a variety of adaptations to restore ER function and ensure cell viability [13].

### 2.1. Activating Transcription Factor 6 (ATF6)

In response to ERS, the transcription factor ATF6 migrates to the Golgi complex. Upon arrival, it undergoes cleavage by two specific proteases, site-1 protease (S1P) and site-2 protease (S2P), which release a transcriptionally active N-terminal fragment known as activating transcription factor 6 protein 50 (ATF6P50) [13,15]. ATF6P50 then acts together with another transcription factor: XBP1s [16]. Once ATF6P50 enters the nucleus, it promotes the transcriptional activity of the genes involved in protein translocation, folding, splicing, and secretion [17]. These genes primarily encode molecular chaperone proteins, which are responsible for assisting in protein transcription. They also encode molecular chaperone proteins in the ER (e.g., BiP), the protein disulfide isomerases (PDIs) involved in the formation of disulfide bonds in proteins, and proteins that constitute the endoplasmic reticulum-associated protein degradation (ERAD) pathway. This pathway aims to restore the impaired folding of ER proteins [18].

### 2.2. Inositol-Requiring Enzyme 1 (IRE1)

IRE1 is a protein that spans the ER membrane. Its N-terminal end faces the lumen of the ER, and its C-terminal end extends into the cytoplasm. Under ERS, IRE1 forms oligomers and autophosphorylates, activating its function as an RNase. This activation leads to the cleavage of XBP1 mRNA, producing activated forms of XBP1s. This process not only helps improve the stability of cellular proteins, but also accelerates the degradation and elimination of misfolded proteins, thereby alleviating the damage caused by ERS [19]. However, under strong or prolonged ERS, IRE1 can interact with TNF receptor-associated factor 2 (TRAF-2), which activates apoptotic pathways, including apoptosis signal-regulated kinase 1 (ASK-1), c-Jun N-terminal kinase (c-JNK), and p38 mitogen-activated protein kinase (MAPK), leading to cell death [13]. The activation of JNK can affect the expression of several apoptosis-related genes, such as BID, BIM, and some members of the BCL-2 family, thereby promoting apoptosis.

### 2.3. Pkr-like Endoplasmic Reticulum Kinase (PERK)

**Figure 1:**
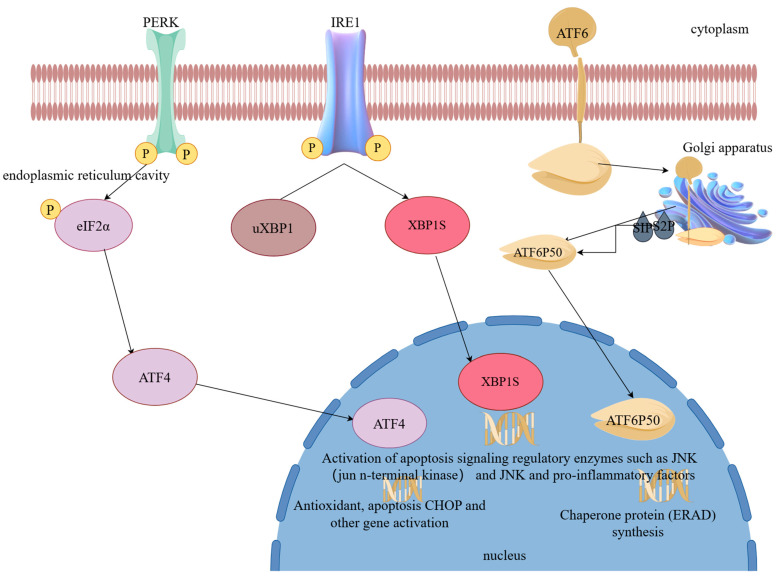
Schematic diagram of the ERS mechanism.

PERK is a member of the ER protein family and belongs to the serine/threonine protein kinase family. Like IRE1, it is a Class I transmembrane protein. When stress is manifested in the ER, PERK dissociates from the chaperone protein BiP/GRP78. This dissociation triggers the autophosphorylation of PERK, thereby initiating a series of signaling processes. PERK activation induces the phosphorylation of eukaryotic translation initiation factor 2*α* (eIF2*α*), which reduces protein synthesis to relieve ERS and also serves as a transcription regulatory molecule [20,21]. Despite a reduction in overall protein synthesis, this phosphorylation specifically increases the translation of ATF4, a transcription factor that plays a role in promoting cell survival and, in some cases, inducing apoptosis. ATF4 also impacts biosynthesis and redox reactions [22]. Under continuous or excessive ERS, PERK activation becomes imbalanced, preventing the cell from returning to its normal state. This situation initiates the PERK-ATF4-CHOP pathway to regulate apoptosis. C/EBP homologous protein (CHOP) is an apoptosis-associated protein that contains two functional domains: the N-terminal transcription activation domain and the C-terminal basic leucine zipper (bZIP) structural domain [23,24]. In the presence of growth arrest and DNA damage-inducible 34 (GADD34), CHOP promotes the dephosphorylation of eIF2α, thereby reversing translational repression. During apoptosis-promoting mRNA translation, proteins are released into the ER, leading to the aggregation of unfolded proteins [25], as shown in Figure 1.

## 3. Endoplasmic Reticulum Stress (ERS) and ERS-Mediated Cell Death

When the body is exposed to adverse environmental stimuli, the ER initiates a stress response and begins to exert its physiological functions. However, when ERS is too severe or prolonged, the homeostasis of the ER can be disrupted, ultimately leading to cell death. Current research on the mechanisms of ERS-induced cell death has mainly focused on autophagy and apoptosis. Nonetheless, researchers have also explored its association with alternative modes of cell death, such as ferroptosis and pyroptosis.

### 3.1. Autophagy

Autophagy is a common intracellular process involved in energy metabolism, cell survival, and the development of many diseases [26]. As a mechanism of cellular self-purification, autophagy plays a key role in maintaining intracellular environmental balance and combating nutritional deficiencies [27]. It is not only an effective way to protect cells from damage, but it also, in some cases, may be involved in the cell death process. Autophagy can be categorized into several types depending on how the materials reach the lysosome, with macroautophagy being the most common. Microautophagy involves the direct degradation of excess cytoplasmic material at the tip of the lysosome [28]. Recent studies have shown that, in addition to these traditional forms of autophagy, there are also selective autophagy-targeting-specific substrates, such as the selective degradation of mitochondria, lipids, or pathogens [29,30]. Autophagy helps reduce ERS by removing misfolded proteins. The relationship between ERS and autophagy activation is well established, with evidence indicating their synergistic ability to efficiently clear misfolded proteins. Atg protein 1 (Atg1) kinase activity increases when the ER is stressed, suggesting that ERS promotes cellular autophagy. Thus, ERS triggers cellular autophagy responses and helps cells adapt to stressful environments [31].

### 3.2. Apoptosis

Apoptosis, a programmed form of cell death, is essential for maintaining normal cell function and homeostasis. It can be initiated through ligand-receptor death pathways, both extracellular and intracellular, thereby ensuring cellular homeostasis. When the ERS response is too intense or persists beyond cellular tolerance levels, it disrupts ER homeostasis, triggering cell death via downstream signaling molecules. While key proteins such as ATF6 and IRE1 do not directly cause cell death, they can activate downstream targets to induce apoptosis. The most intensively studied molecules in this pathway are CHOP and caspase-12 [32]. CHOP is a transcription factor that regulates apoptosis and plays a particularly important role in apoptosis induced by ERS. Normally, CHOP is expressed at very low levels; however, under ERS, CHOP expression increases significantly. Caspase-12, a member of the caspase family localized to the ER membrane, plays a specific role in ERS-mediated cell death [33]. Under normal conditions, caspase-12 remains inactive in the ER and is activated in response to ERS, such as through calcium ion imbalance or the accumulation of unfolded proteins. Activation of caspase-12 is essential for the apoptotic process mediated by ERS and leads to further activation of the caspase pathway in the cytosol through caspase-9, leading to cell death [34]. Activation of caspase-12 under ERS conditions may involve the action of the calcium-activated proteases calpain and TRAF-2. Normally, TRAF-2 forms a complex with inactivated caspase-12, and ERS cleaves and activates caspase-12 through TRAF-2, initiating the apoptotic pathway [35].

### 3.3. Ferroptosis 

Ferroptosis is an emerging mode of cell death [36]. The accumulation of iron ions (Fe^2+^) due to abnormalities in intracellular iron metabolism triggers the Fenton reaction, which produces a large number of lipid peroxides, ultimately leading to cell death [37]. The cystine/glutamate antiporter (System xc^−^) and glutathione peroxidase 4 (GPX4) are key factors in the regulation of ferroptosis. When System xc^−^ or GPX4 is inhibited, intracellular lipid peroxides accumulate, serving as an important signal of ferroptosis [38,39]. In addition, the p53 gene, a well-known tumor suppressor, decreases the expression of solute carrier family 7 member 11 (SLC7A11), contributing to iron accumulation and, consequently, cell death [40]. It has been found that reducing the expression of the cellular stress response factor ATF4 diminishes System xc^−^ activity and makes human glioma cells more susceptible to ferroptosis, suggesting that the ATF4-System xc^−^ pathway may be an additional mechanism by which ERS affects ferroptosi. Ferroptosis and ERS have been observed in various disease models, such as ulcerative colitis; in these cases, the use of certain PERK inhibitors has been shown to significantly inhibit this form of cell death [41]. Cigarette smoke condensate triggers ferroptosis in human bronchial epithelial cells and activates the PERK pathway in response to ERS. In addition, ferroptosis is associated with ischemia-reperfusion injury (IRI) in diabetic myocardia and is linked to the ERS pathway [42]. In summary, ferroptosis emerges as a consequence of dysregulated iron metabolism and the accumulation of lipid peroxides, which is closely related to abnormalities in the cellular antioxidant system and ERS.

### 3.4. Pyroptosis

Cellular pyroptosis is a recently unveiled mechanism of programmed cell death. It is mainly characterized by the swelling of the nucleus and cytoplasm and the activation of the caspase family of enzymes, which ultimately leads to the rupture of the cell membrane and the release of pro-inflammatory cytokines such as interleukin 1*β* (IL-1*β*) and interleukin-18 (IL-18). This cascade triggers inflammation and leads to cell death [43,44]. In the classical activation pathway, caspase-1 and its synergistic inflammasome, nucleotide-binding oligomerization domain, leucine-rich repeat, and pyrin domain-containing 3 (NLRP3), play key roles. This complex system is composed of NLRP3, procaspase-1, and apoptosis-related speck-like protein containing a CARD (ASC), which contains structural domains that recruit caspase [45]. Cigarette smoke condensate induces ferroptosis in human bronchial epithelial cells and activates the PERK pathway in response to ERS. In addition, ferroptosis is associated with IRI in diabetic myocardia and is linked to the ERS pathway [46]. It has been found that ERS inhibitors, such as taurine, can exert a protective effect by reducing the level of IRE1*α* phosphorylation, decreasing the concentration of tumor necrosis factor-*α* (TNF-*α*) and inhibiting NLRP3 inflammasomes, thereby preventing cellular pyroptosis [47]. Such intervention protects beta cells from the islet dysfunction induced by inorganic arsenic [48]. Similarly, the application of the pharmacological inhibitor STF-083010 or knockout of the XBP1 gene can specifically inhibit the IRE1/XBP1 pathway and significantly reduce cadmium-induced NLRP3 inflammasome activation and cellular pyroptosis [49]. In summary, the modulation of intracellular stress responses and inflammatory signaling pathways provides a feasible strategy to reduce the occurrence of pyroptosis and thereby protect cells from injury.

## 4. Renal Fibrosis

The kidney is a vital organ of the human body. It is not only responsible for processing the body’s metabolic waste and controlling the secretion and reabsorption of substances, but also for regulating the acid–base balance of the blood. Despite their small size, the kidneys process a large volume of blood. They are also part of the endocrine system as they secrete hormones such as renin and erythropoietin (EPO) and serve as targets for hormones such as aldosterone, antidiuretic hormone, and natriuretic peptides.

### 4.1. Causes of Renal Fibrosis

The inflammatory response is associated with autoimmune diseases, diabetes, alcohol consumption, and other unhealthy habits. Many of these factors work together to further exacerbate renal fibrosis [50,51]. When the kidney is damaged by immune or non-immune factors, an inflammatory response occurs, which accelerates renal regeneration and repair [52]. Inflammatory cells and the renal lamina propria are involved in both inflammatory and fibrotic processes during renal injury. The pro-fibrotic and pro-inflammatory responses of inflammatory cells play a key role in renal injury. Early studies have revealed that renal tissues contain a large number of inflammatory cells that secrete a plethora of pro-fibrotic factors and cytokines, exacerbating the inflammatory response and ultimately causing renal fibrosis. Chronic inflammation and cytokines promote proliferation, differentiation, epithelial-mesenchymal transition (EMT), and interstitial cell activities, as well as the proliferation and aggregation of fibroblasts, which are critical events in the pathogenesis of renal fibrosis. Moreover, extracellular matrix production accelerates renal fibrosis [53].

### 4.2. Mechanisms of Renal Fibrosis

#### 4.2.1. Effects of TGF-*β*

Transforming growth factor *β* (TGF-*β*) is a growth factor that is widely expressed in the human body. It encompasses a number of family members involved in the regulation of cell growth, development, and differentiation, and it plays a crucial role in ligand secretion and the activation of the extracellular matrix and pre-signaling pathways. TGF-*β* is a polypeptide with multiple biological activities, promoting cell proliferation, inducing cell differentiation, and potently inhibiting apoptosis. The TGF-*β*/sterile alpha motif domain (Smad) signaling pathway is important, with Smad proteins serving as key regulatory molecules closely related to cell proliferation and differentiation [54]. TGF-*β*1, in particular, emerges as a key regulatory molecule in renal fibrosis, orchestrating downstream signaling cascades. In this regulatory framework, Smad3 translocates into the cell nucleus upon interaction with Smad4, leading to reduced DNA methylation and acetylation, as well as inducing the transcription of miRNAs involved in renal fibrosis [55,56,57].

#### 4.2.2. Effects of the Wnt Signaling Pathway

The Wingless/Integrated (Wnt) signaling pathway is an important extracellular regulatory signal present in various tissues and organs. It plays a key regulatory role in cell growth, proliferation, differentiation, and the maintenance of a stable intracellular microenvironment. This signaling pathway is crucial in regulating tissue structure and function and in maintaining normal growth and development [58]. *β*-catenin, a protein localized to the cell surface, is central to this pathway. It regulates its distribution across the cell membrane and plays a pivotal role in the Wnt signaling pathway [59].

## 5. Endoplasmic Reticulum Stress in Renal Fibrosis

### 5.1. Activating Transcription Factor 6 (ATF6)

ATF6 is one of the three branches of the UPR pathway [16]. Activation of ATF6 leads to alterations in the lipid metabolism and, subsequently, to lipotoxicity and fibrosis. However, the phenotype of lipotoxicity-induced fibrosis is significantly attenuated after ATF6 knockdown [60]. Progressively higher levels of advanced oxidation protein products (AOPP) are observed in patients with kidney disease, and AOPP accumulation can exacerbate renal fibrosis. AOPPs promote the expression of epithelial cell markers, as well as markers of ERS through multiple signaling pathways, including PERK, ATF6, and IRE1. The ATF6 pathway also plays an important role in AOPP-induced hypertrophy and in the EMT of human proximal renal tubular epithelial cells (HK-2 cells) [61].

### 5.2. Xbox Binding Protein 1 (XBP1)

The activation of IRE1*α* eventually leads to the formation of spliced XBP. It has been shown that, after acute kidney infarction, the expression of XBP1 decreases, exhibiting a negative correlation with the degree of renal injury. XBP1 knockout mice exhibit more intense renal damage compared to normal mice [62]. The inactivation of XBP1 causes renal interstitial inflammation and associated fibrosis, leading to a gradual decline in kidney function over several months. In vivo, XBP1 expression can fully restore chronic kidney injury following the activity of XBP1 or IRE1*α* [63]. ERS promotes ferroptosis-associated EMT progression via activation of the XBP1-Hrd1-Nrf2 pathway. When ERS is continuously activated, the upregulation of Hrd1 via this pathway coordinates the destruction of misfolded proteins, causing the downregulation of Nrf2, which, in turn, stimulates EMT [64].

### 5.3. C/EBP-Homologous Protein (CHOP)

CHOP, a member of the C/EBP family of transcription factors, is implicated in signaling events associated with stress-induced ER apoptosis [65]. Rats lacking the CHOP gene have exhibited markedly reduced interstitial fibrosis compared to rats with the gene [66]. The formation of autophagic vesicles by unilateral ureteral occlusion (UUO) and the increased expression of autophagy-associated proteins Beclin-1 and p62 in renal tissues have also been reported. However, these UUO-induced changes are notably diminished in CHOP knockout mice, suggesting a key role for CHOP in the progression of renal fibrosis, possibly through the regulation of autophagy and apoptosis [67]. Autosomal dominant tubulointerstitial kidney disease due to uromodulin (ADTKD-UMOD) is an inherited kidney disease characterized by mutations in the UMOD gene with an autosomal dominant mode of inheritance. When cysts occur, they can also cause a considerable amount of pericapsular fibrosis [68]. Damage to the endoplasmic reticulum also plays a key role in the pathology of ADPKD [69]. Individuals with ADTKD-UMOD show significant co-localization of UMOD proteins with glucose-regulated GRP78 and CHOP. In vitro experiments have demonstrated that ERS significantly increases CHOP expression and induces the conversion of renal tubular epithelial cells to myofibroblasts. The knockdown of CHOP restores the aforementioned expression. Thus, CHOP-specific activation mediated by the UMOD protein or its processing through the GRP78/CHOP pathway plays a crucial role in this context [70].

### 5.4. Inositol-Requiring Enzyme 1 (IRE1)

In the presence of adaptive ERS, the interaction of IRE1*α* with BiP is attenuated, leading to the further activation of IRE1α via oligomerization and autophosphorylation [71]. It has been reported that streptozotocin (STZ)-specific IRE1 knockout mice have significantly larger fibronectin-positive areas than STZ-treated control mice. Additionally, the podocytes from STZ-treated IRE1 knockout mice exhibit stronger pedicel effacement and less extracellular matrix (ECM) deposition than podocytes from STZ-treated control mice [72]. In the I/R model, compared with the control group, the mRNA expression of fibronectin (FN) and collagen IV (Col IV) proteins, as well as FN and COL4A1 mRNA, was significantly increased in the I/R group at 4, 8, and 12 weeks after reperfusion. In contrast, when injected with Irestatin 9389 (an IRE1 inhibitor), the proteins of Col IV and FN and the mRNA levels of COL4A1 and FN were significantly reduced in the I/R + Irestatin 9389 group [73].

## 6. Amelioration of Renal Fibrosis by Modulation of Endoplasmic Reticulum Stress

### 6.1. Drugs

Dapagliflozin

Dapagliflozin is a sodium-glucose cotransporter 2 (SGLT-2) inhibitor that is already used in clinical practice. In patients with type 2 diabetes, SGLT2 inhibitors provide benefits such as reducing the risk of hyperglycemia, facilitating weight loss, and lowering blood pressure [74]. In addition, clinical studies have demonstrated the nephroprotective and cardioprotective effects of SGLT2 inhibitors in type 2 diabetes [75,76]. In a study employing a rat model with diabetic kidney disease (DKD), the animals were randomly divided into groups, where half of them received dapagliflozin treatment. The results showed that dapagliflozin significantly inhibited reactive oxygen species (ROS) levels and ERS in diabetic rats. It has been hypothesized that the protective effect of dapagliflozin against DKD may be related to the inhibition of ERS [77]. Furthermore, in a UUO model, rats administered dapagliflozin daily for 7 days exhibited attenuation of mitochondrial dysfunction and ERS induced by UUO. Dapagliflozin was shown to ameliorate renal fibrosis by inhibiting receptor-interacting protein 1 (RIP1), receptor-interacting protein 3 (RIP3), and mixed lineage kinase domain-like (MLKL)-mediated necroinflammation, as well as ERS mediated by the Wnt3*α*/*β*-catenin/glycogen synthase kinase-3*β* (GSK-3*β*) signaling pathway in UUO [78].

Ginsenoside Rg1

Ginsenoside Rg1, a steroidal saponin found in high amounts in ginseng, has been shown to possess anti-inflammatory and anti-aging properties in a variety of animal models [79]. It has been shown that Rg1 can slow the progression of CsA nephropathy by inhibiting the renal tubular epithelial apoptosis induced by ERS [80]. By reducing PERK1/2 expression, Rg1 has also been shown to inhibit epithelial-to-myofibroblast conversion [81]. In addition, Rg1 has been shown to attenuate senile renal mesangial fibrosis by inhibiting ERS and apoptosis [82]. Bavachin (BV) induces increased levels of oxidative stress in tubular epithelial cells and triggers BiP/eIF2*α*/CHOP-mediated ERS, whereas Rg1 inhibits BV-induced ERS. As a result, BV-induced EMT and renal fibrosis are alleviated. Therefore, Rg1 is a promising therapeutic agent [83].

Shenkang Injection

Shenkang injection (SKI) (Z20040110) is a new anti-CKD drug that has been commonly used in Chinese clinics for more than 20 years [84]. SKI has been reported to attenuate tubulointerstitial fibrosis (TIF) in vivo by blocking pericyte-myofibroblast transformation in the kidneys of rats with obstructive nephropathy [85]. In DKD mice, various markers of renal tubular EMT, including E-adhesin, collagen I, vimentin, and α-SMA, were elevated in association with GRP78, a marker of ERS. After the use of SKI, these indicators were reduced to varying degrees. Furthermore, it has been demonstrated that SKI alleviates renal injury through the PERK-eIF2α-ATF4-CHOP signaling pathway [86].

### 6.2. Physiological Regulator

CORM-2

Carbon monoxide (CO) is an important metabolite of heme with protective effects against a variety of diseases in several animal models [87,88]. Carbon monoxide releasing molecule-2 (CORM-2) has been found to have inhibitory effects on the ERS in pancreatic islets and vascular endothelial cells [89]. Additionally, CO has exhibited protective effects against obstructive renal fibrosis in mice [90]. Pretreatment with CORM-2 successfully reduces lipopolysaccharide (LPS)-induced oxidative stress, ERS-induced inflammation, and cell death during AKI. These findings suggest that CORM-2 may be a potential therapeutic option for ERS-mediated AKI [91].

### 6.3. Protein

Renalase

Renalase is a flavoprotein with redox activity that acts as an antioxidant and has a protective effect against several human kidney diseases in a number of animal models [92,93]. Moreover, emerging evidence suggests that renalase might also confer protective benefits to the human heart [94]. In a study employing a unilateral mouse model of complete ureteral obstruction, the effects of renalase on renal fibrosis were investigated utilizing a HK-2 cell model induced by TGF-*β*1. The findings revealed the activation of ERS in models of induced renal fibrosis. Remarkably, renalase supplementation alleviated renal fibrosis and suppressed ERS [95].

### 6.4. Others

Chitosan

Chitosan (COS) is a class of naturally occurring oligomers composed of *β*-(1,4)-D-glucosamine with a degree of polymerization of 2–8 [96]. Recently, chitosan has been found to have therapeutic effects in various diseases such as obesity, diabetes, Alzheimer’s disease, and non-alcoholic fatty liver disease (NAFLD) [97,98]. The nephroprotective properties of chitosan have been well studied and its efficacy has been confirmed, albeit only in animal models. Using an IRI model to simulate AKI, COS has demonstrated the ability to reduce IRI-induced AKI and protect glomerular filtration function. This occurs by modulating pathways such as oxidative stress, mitochondrial damage, and ERS. These findings provide new ideas for the treatment of AKI [99].

Melatonin

Melatonin, a hormone secreted by the pineal gland, plays an important role in regulating the biological clock [100]. Alongside its fundamental function, melatonin exhibits notable anti-inflammatory and anti-apoptotic effects [101,102]. Unfortunately, these effects have also only been reported in animal models. In an experiment employing a randomized allocation method, rats were divided into sham operation, ischemia-reperfusion (I/R), and ischemia-reperfusion + melatonin groups. The results showed that melatonin administration alleviates cytolysis and lipid peroxidation, as well as improves renal function and morphology, compared with the I/R group. Additionally, melatonin significantly reduces ERS-related factors such as GRP78, XBP1, ATF6, and CHOP [103].

Coenzyme Q10

Coenzyme Q10 (CoQ10) is an endogenous lipid-soluble component containing a benzoquinone ring with 10 isoprenoids on the side chain. CoQ10 acts as a diffusible electron transporter for the mitochondrial respiratory chain [104]. The renoprotective effects of CoQ10 have been demonstrated in a variety of animal models [105,106]. In the UUO rat model, UUO leads to the evolution of renal fibrosis, and oxidative stress induces excessive ERS and mitochondrial dysfunction. However, fibrosis, ERS, and mitochondrial dysfunction were found to be relieved after CoQ10 treatment. Furthermore, CoQ10 was found to inhibit necroinflammation in UUO by suppressing the Wnt3*α*/*β*-catenin/GSK-3*β* signaling-mediated RIP1, RIP3, and MLKL pathways [107]. As shown in Table 1.

## 7. Conclusions and Outlook

In recent years, there has been a surge in research on ERS for the treatment of renal fibrosis. Many drugs, including traditional Chinese and Western medicines, as well as molecularly targeted agents, have been used to modulate ERS and reduce renal fibrosis. Examples include dexmedetomidine, apelin-13, and ginsenoside-Rg1, among others. The most prominent of these are tauroursodeoxycholic acid (TUDCA) and 4-phenylbutyric acid (4-PBA). It has been reported that 4-phenylbutyrate inhibits clindamycin-induced AKI by inhibiting CHOP/GADD153 [127]. TUDCA-treated, CKD-derived hMSCs improve therapeutic efficacy in ischemic disease via PrPC [128]. These substances enhance the organ’s protein folding capacity and delay end-stage renal disease (ESRD), which may protect the kidney from fibrotic damage [129,130]. ERS-related drugs such as alginate and taurine are currently undergoing clinical investigation for their efficacy in combating Alzheimer’s disease and amyotrophic lateral sclerosis [131]. Although we have made remarkable progress and gained a deeper understanding of the role of ERS in regulating cell fate, tissue damage, and a wide range of diseases, many unresolved questions remain that require further research. Furthermore, the current knowledge of the involvement of ERS and the UPR in human diseases primarily stems from animal models, which poses limitations on our comprehension of their relevance in human pathology. Currently, commercially available drugs targeting the ERS do not act directly on regulatory sites upstream of the ER. Therefore, further studies in renal fibrosis patient samples and clinical trials are needed to ascertain the precise modulatory effects of ERS-targeting drugs in renal fibrosis. Additionally, using the ERS pathway for disease treatment represents a new strategy for precision medicine, offering promising perspectives and opportunities for the treatment of many refractory diseases. Continued exploration in this field holds immense potential for advancing therapeutic interventions across a spectrum of challenging clinical conditions.

## Figures and Tables

**Table 1:** Impact of endoplasmic reticulum stress on renal fibrosis.

	Name	Animal/Cell Model	Target Pathway	Target Protein	References
Drugs	Cyproheptadine	STZ-induced mice	NR	CHOP, p-eIF2*α*	[108]
	Dapagliflozin	DIO Wistar mice	NR	CHOP, GRP78	[77]
		UUO mice	Wnt 3 *α*/*β*-catenin/ GSK-3*β*	CHOP, IRE1*α*	[78]
	Ginsenoside Rg1	(SAMR1) mice	NR	CHOP, p-PERK/PERK	[82]
		BV indeed zebrafish/human renal tubular epithelial	BiP/eIF*α*/CHOP	BiP, CHOP	[83]
	Withaferin A	UUO mice	NR	GRP78, GRP94, ATF4, CHOP, eIF2*α*,	[109]
	Shenkang injection	DKD mice	PERK-eIF 2 *α*-ATF4-CHOP	GRP78	[86]
	Quercetin	CdCl2-treated mice	NR	CHOP	[110]
	Dexmedetomidine	IRI mice	*α*2AR/PI3K/AKT	GRP78, CHOP	[111]
	LCZ696	UUO mice	ASK1/JNK/p38 MAPK	CHOP, IRE1*α*	[112]
	Salvianolic acid A and B	UUO mice	PDGF-C/PDGFR-*α*	CHOP, GRP78	[113]
	MS-275	LPS-induced mice	NR	CHOP, GRP78	[114]
	Endothelin-Converting Enzyme Inhibitor	Adenine diet-induced mice/HK-2 cell	NR	IRE1*α*	[115]
Physiological regulator	Ghrelin	UUO mice	NR	CHOP, caspase 12	[116]
	CORM 2	LPS-induced AKI	NR	sXBP1, GRP78	[91]
	Interleukin-10	UUO mice	NR	PERK, eIF2*α*, IRE1*α*, XBP1, ATF6*α*	[117]
	Apelin-13	Iohexol-induced CI-AKI model/HK-2 cell	NR	CHOP, GRP78	[118]
Protein	Renalase	UUO mice/TGF-*β*1-induced fibrotic HK-2 cells	GSK-3*β*/Snail	CHOP, PERK, ATF 4	[95]
	Calcium binding protein-D28 k	db/db diabetic mice	NR	CHOP, GRP78, IRE1*α*	[119]
	Ulinastatin	LPS-induced HK-2 Cells	TLR4/NF-*κ*B and Nrf2/HO-1	CHOP, GRP78, IRE1*α*	[120]
	SIK2	db/db diabetic mice	NR	eIF2*α*, CHOP	[121]
	C-phycoerythrin	HgCl2-induced AKI	NR	IRE1*α*, PERK, PERK	[122]
Others	Chitosan	I/R-induced AKI	NR	CHOP, GRP78.	[99]
	Silymarin	UUO mice	NR	PERK, CHOP, caspase-3	[123]
	Melatonin	I/R-induced AKI	AKT	GRP78, p-PERK, XBP1, ATF6, CHOP, JNK	[103]
	Farnesoid X receptor	Uninephrectomy	NR	CHOP, GRP78.	[124]
	Crocin	db/db mice	PI3K/AKT/Nrf2	CHOP, GRP78	[125]
	Coenzyme Q10	UUO mice	Wnt3*α*/*β*-catenin/GSK-3*β*	CHOP, IRE1*α*, XBP1S	[107]
	EW-7197	db/db diabetic mice	NR	ATF6	[126]

NR: not reported.
